# Supplementary material for: Patterns of Intron Gain and Loss in Fungi
Source: PLoS Biol. 2004 Nov 30;2(12):e422. doi: 10.1371/journal.pbio.0020422 (PMC532390; doi:10.1371/journal.pbio.0020422)
Supplement: Table S1 — Also available at http://genes.mit.edu/NielsenEtAl/. (4.3 MB ZIP). [file pbio.0020422.st001.zip › NielsenEtAl/html/1316.html]

AN7401.1.NCU04997.1.MG09792.1.FG06445.1


```
 CLUSTAL W (1.82) Multiple Sequence Alignments - Introns Inserted


Sequence 1: MG09792.1	431 aa
Sequence 2: FG06445.1	367 aa
Sequence 3: AN7401.1	355 aa
Sequence 4: NCU04997.1	506 aa
Alignment Length: 544 aa
Number Identitical Residues: 87 aa
Alignment Score (without introns) 6448


MG09792.1 	MRSASFLPAFIGALLP---TAN~---~----AQACGLNQVAQAAGLQYFGTAVGE-GAVN
NCU04997.1	---MHLPTVLSVIFAAAGVT--~---~-------AQLNDLAVAAGLKYFGTAGQEGTITS
FG06445.1 	MR-FSILPTVALALYNGLVAAN~PVP2ELESRQTSGLDAAMKAAGKKYFGTAL---TVRN
AN7401.1  	---MFKLHSFTAALAAGLLTTI2---~----VSAAGLHEAALAAGLEYFGTATDNGELT-
          	         .   :  .  ::           .:. *.    *** :*****  ..    

MG09792.1 	EQPYMNIVNNINEFGQVVPENGQK~WQSTQPNRGQFSYTQGDIVTNVATRNNQVLRCHTL
NCU04997.1	DTTYRNIINNVHEFGQLTPENGMK~WDATEGQPGKFTFQSGDIVAGVAKSNGQLLRCHTL
FG06445.1 	DAGETNVLNTKGEFGSITPENAMK~WEAIQPNRGQFNWGPADQHANAATQRGMELRCHTL
AN7401.1  	DIPYVTQLNNTADFGQITPGNTQK0WDSTEPSQGTFSFTKGDVIADLADANGQYLRCHTL
          	:    . :*.  :**.:.* *  * *:: : . * *.:  .*  :. *  ..  ******

MG09792.1 	VWHSQLPGWV1SSGSWNRQSLEQVIVTHINNVMGHYLGKCYAWDVVNEA~IDDN--GGWR
NCU04997.1	VWYSQLPSWV1SNGSWNRNTLQSVIESHISNVMGHYKGQCYAWDVVNEA~ISDDAQGNYR
FG06445.1 	VWHSQLPSWV~ANGNWNNQTLQQVMKDHINAVMGRYKGKCTHWDVVNEA1LNED--GTYR
AN7401.1  	VWHNQLPSWV1TSGSWTNTTLTAALRNHITNVVNHYKGRCIHWDVVNE-~----------
          	**:.***.** :.*.*.. :*  .:  **. *:.:* *:*  ******            

MG09792.1 	DSVFYRVFGTDFIPLSFKAAKAADPNTKL2YYNDYNLE~YNQAKTDRAVELVKIVQAAGA
NCU04997.1	DSIFFKTFKTDYFPIAFNAAKKADPNAKL2YYNDYNLE~YNGAKTERALELVKLIKAAGA
FG06445.1 	DSVFYRVIGEAFIPIAFRMALAADPTTKL~YYNDYNLE~YGGAKTAGAIRITKLIQSYGL
AN7401.1  	-----------------------------~-------E1YGGAKAAGARAIVQLIKNAGV
          	                                     * *. **:  *  :.::::  * 

MG09792.1 	PIDAVGLQGHMIVGSTP-------SRSALTTMLRRFTALNVDVSYTEVDIRHSSLPASSQ
NCU04997.1	PIDGVGFQAHMTVGGTP-------SRSAMATLLKRFTALGVEVAYTELDIAHKNQPSSSS
FG06445.1 	RIDGVGLQAHMTSESTPTQSTVTPSRANLASVLNSFTKLNVDVAYTELDIRMN-TPANQQ
AN7401.1  	KIDGVGFQAHFSVGTVP-------SRSSLASVLQSFTSLGVEVAYTEADVRIQ-LPTSAT
          	 **.**:*.*:    .*       **: ::::*. ** *.*:*:*** *:  .  *:.  

MG09792.1 	AAAQQGVDYANMVGSCLDVGPRCVGFTVW~GFTDKHSWIPS--------------TFPGT
NCU04997.1	VQAQQATDYANMVGSCVDVD-GCVGVTIW~GFIDKYNWIQNGNAAIFDSSFNKKPAYSAI
FG06445.1 	KLQANAAAYARMVGSCMDVK-RCVGVTVW0GISDKYSWVPG--------------TFPGE
AN7401.1  	TLAQQSTDFQNLAGSCVDTA-GCVGFTIW~DWTDKYSWVPS--------------TFSGY
          	    :.. : .:.***:*.   ***.*:* .  **:.*: .              ::.. 

MG09792.1 	GDAMLYDANYNKKPAWSS-----VSSVLRA~KATGSGCGPATNLPTTTVATTSPPTSFTT
NCU04997.1	SSVLAAAATKAPTPAKNANSPPAVHTTLRT~AIATAPACEATATAPKATAAP--------
FG06445.1 	GSALLWDDNFNKKPSYTS-----SLNTIRA1------CWKCHRLLVSIS-----------
AN7401.1  	GAALPWDENFVKKPAYDG-----LLVGLGG~TVTTTTTTTATSTTTSATATS--------
          	. .:    .   .*:  .         :     : :    .     .  ::.        

MG09792.1 	SVTSTTTTPPPQQTTPPVTNPGTVPQWQQCGGINWQGGTVCVSPFRCVKLNDWYYQCL~A
NCU04997.1	-------AAQPAKPATGSSTGAQQPRWAQCGGNGYTGPTACQAPYTCKVQNPWYSQCF2G
FG06445.1 	----------------------------------------------------------~-
AN7401.1  	-------TAT-------------SPHWGQCGGIGWTGPTLCASPWTCTYVNDWYSQCL~-
          	       :.               .   .... .  . : . :.  .   .    ..   

MG09792.1 	------------------------------------------------------------
NCU04997.1	TDRSYLGTATSRDAKIDSRERMEKQRKESTRTAAGQLEGKKEKGMAEPGIRGYLPAWPRN
FG06445.1 	------------------------------------------------------------
AN7401.1  	------------------------------------------------------------
          	                                                            

MG09792.1 	--------------
NCU04997.1	ESSWEAAVDGKHTL
FG06445.1 	--------------
AN7401.1  	--------------
          	
```
